# piRNA-823 Is Involved in Cancer Stem Cell Regulation Through Altering DNA Methylation in Association With Luminal Breast Cancer

**DOI:** 10.3389/fcell.2021.641052

**Published:** 2021-03-15

**Authors:** Xin Ding, Ya Li, Jinhui Lü, Qian Zhao, Yuefan Guo, Ziye Lu, Wenjing Ma, Pengfei Liu, Richard G. Pestell, Chunli Liang, Zuoren Yu

**Affiliations:** ^1^Research Center for Translational Medicine, Shanghai East Hospital, Tongji University School of Medicine, Shanghai, China; ^2^University College London, London, United Kingdom; ^3^Pennsylvania Cancer and Regenerative Medicine Research Center, Baruch S. Blumberg Institute, Doylestown, PA, United States; ^4^Dalian Medical University, Dalian, China

**Keywords:** piR-823, cancer stem cell, breast cancer, DNA methylation, non-coding RNAs

## Abstract

Cancer stem cells (CSCs) are believed to be the main source of cancer relapse and metastasis. PIWI-interacting small non-coding RNAs (piRNAs) have been recently recognized to be relevant to cancer biology. Whether and how piRNAs regulate human CSCs remain unknown. Herein, upregulation of piR-823 was identified in tested luminal breast cancer cells, especially in the luminal subtype of breast CSCs. Enforced expression or targeted knockdown of piR-823 demonstrated its oncogenic function in regulating cell proliferation and colony formation in MCF-7 and T-47D breast cancer cells. In addition, piR-823 induced ALDH (+) breast CSC subpopulation promoted the expression of stem cell markers including OCT4, SOX2, KLF4, NANOG, and hTERT, and increased mammosphere formation. Tail vein injection of magnetic nanoparticles carrying anti-piR-823 into the mammary gland of tumor-burdened mice significantly inhibited tumor growth *in vivo*. DNA methyltransferases (DNMTs) including DNMT1, DNMT3A, and DNMT3B were demonstrated to be the downstream genes of piR-823, which regulate gene expression by maintaining DNA methylation. piR-823 increased the expression of DNMTs, promoted DNA methylation of gene adenomatous polyposis coli (APC), thereby activating Wnt signaling and inducing cancer cell stemness in the luminal subtype of breast cancer cells. The current study not only revealed a novel mechanism through which piRNAs contribute to tumorigenesis in breast cancer by regulating CSCs, but also provided a therapeutic strategy using non-coding genomes in the suppression of human breast cancer.

## Introduction

In the last few decades, non-coding RNAs (ncRNAs) have been demonstrated to play important roles in regulating gene expression and biological processes. In particular, long ncRNAs (lncRNAs), circular ncRNAs (circRNAs), and small ncRNAs including microRNAs (miRNAs), P-element-induced wimpy testis (PIWI)-interacting RNAs (piRNAs), and small interfering RNAs (siRNAs) are involved in the regulation of cancer development and progression mostly by regulating gene expression at the transcriptional and post-transcriptional levels *via* RNA-RNA or RNA-DNA or RNA-protein interactions (Anastasiadou et al., 2017; Romano et al., 2017). Among these ncRNAs, miRNAs, lncRNAs, and circRNAs have been widely investigated and well confirmed to be important regulators in diverse cancer types (Yu and Pestell, 2012; Wang et al., 2016; Anastasiadou et al., 2017; Romano et al., 2017; Chen and Huang, 2018). However, the function of piRNAs, which were first identified in the testis as molecules that are 24–32 nt in length, in tumorigenesis is poorly understood. piRNAs have been considered as germ cell-specific small RNAs binding to PIWI proteins and functioning in stemness maintenance, transposon silencing, epigenetic modification, and post-transcriptional regulation of gene expression, thereby maintaining genome stability during germ line development and spermatogenesis (Girard et al., 2006; Story et al., 2019; Sato and Siomi, 2020).

Emerging evidence shows the presence of a subset of piRNAs with aberrant expression in tumor cells (Martinez et al., 2015; Lü et al., 2020). Our previous work (Lü et al., 2020) demonstrated the expression of PIWIL2, but not PIWIL1, in human breast cancer cells. A total of 415 piRNA sequences were identified from the secretome of MCF-7 cells (Lü et al., 2020). Consistent with these findings, a high-throughput deep sequencing analysis identified a group of small RNA sequences matching piRNAs in breast cancer (Hashim et al., 2014). A recent study found that piRNA-36712 was downregulated in breast tumor tissues compared with healthy controls, correlating with poor outcome in breast cancer patients (Tan et al., 2019). The complex of piR-932 and PIWIL2 was reported to promote methylation of the promoter region CpG island of the latexin gene, altering latexin expression, and thereby blocking breast cancer metastasis (Zhang et al., 2013). These piRNAs showed altered expression in cancer cells, especially in cancer stem cells (CSCs). Although piRNAs are supposed to regulate tumorigenesis and tumor progression by epigenetic regulation at the genome DNA level and/or gene translation at the message RNA level, the mechanisms regulating CSCs are yet to be determined.

Breast cancer is one of the most prevalent malignancies and the major cause of cancer-related death in women all over the world (Veer et al., 2002; Forouzanfar et al., 2011). It is generally classified into four subtypes including luminal, Her2+, basal-like, and normal-like upon the expression patterns of progesterone receptor (PR), estrogen receptor (ER), and human epidermal growth factor receptor 2 (Her2) (Odle, 2017). Although great advances have been achieved in the therapeutics of human breast cancer, tumor recurrence and metastasis are still incurable, mainly due to the therapy-resistance of a small population of stem-like cancer cells called breast CSCs or tumor initiating cells (TICs). Lin^–^CD24^–/low^CD44^+^ or ALDH + cell subpopulations have been well-validated as CSCs in human breast cancer tumors (Al-Hajj et al., 2003; Ginestier et al., 2007), which are characterized by self-renewal, differentiation, and have the strong capability to regenerate tumors after transplantation *in vivo*. The aberrant expression of PIWI proteins and piRNAs in breast CSCs have been recently reported (Amaar and Reeves, 2020; Lü et al., 2020). The regulation of CSC expansion by piR-016658 and piR-016975 was demonstrated in our previous study (Lü et al., 2020). Targeting piRNAs in suppressing CSCs may provide a novel strategy to treat cancer patients, especially in the prevention of CSC-induced drug resistance and cancer relapse.

Herein, overexpression of piRNA-823 was first observed in the luminal subtype of breast cancer, as well as in ALDH + breast CSCs. piRNA-823 promoted cell proliferation, colony formation, and the tumor regenerative abilities of both MCF-7 and T-47D breast cancer cells. In addition, piRNA-823 induced stemness and expansion of breast CSCs by regulating DNA methylation and activating Wnt signaling. A nanoparticle-based gene therapy targeting piRNA-823 efficiently inhibited tumorigenesis and tumor growth in a mice xenograft model transplanted with MCF-7 cells.

## Materials and Methods

### Human Breast Tumor Samples

Human breast cancer samples were collected from Tongji University Shanghai East Hospital. All the procedures were approved by the Institutional Review Board (IRB) of Tongji University Shanghai East Hospital. All patients were provided with a written informed consent form.

### Animals

Four to six-week-old female nude mice were purchased from the Silaike Animal Company (Shanghai, China). All animal procedures were approved by the Institutional Animal Care and Use Committee of the Tongji University School of Medicine.

### Cell Lines and Cell Culture

All breast cancer cell lines, originally obtained from the American Type Culture Collection (ATCC), were maintained in our lab, and cultured at 37°C with 5% CO_2_ in Dulbecco’s Modified Eagle’s Medium (DMEM) medium supplemented with 10% fetal bovine serum (Gibco) and 1% penicillin–streptomycin.

### Oligos and Transfection

All oligos for piRNA mimics or antisense inhibitors were synthesized by GenScript (Nanjing, China). The sequence for the piR-823 mimic is: 5′ r(AGCGUUGGUGGUAUAG UGGUGAGCAUAGCUGC)dTdT 3′ (double-strand); for anti-piR-823 is: 5′ G^∗^C^∗^AGCUAUGCUwCACCACUAUACCACC AAC^∗^G^∗^C^∗^U^∗^ (2-O-Methyl to all bases). Oligo transfection was performed using RNAiMAX (Invitrogen) following the manufacturer’s instructions. A final concentration of 30 nM was used in all *in vitro* assays.

### Real-Time PCR Analysis of piRNA

RNA extraction, small RNA reverse transcription, and piRNA real-time PCR analysis were performed following the procedure described in our previous publication (Lü et al., 2020). The sequence for the piR-823 primer is: 5′ AGCGTTGGTGGTATAGTGGT 3′.

### ALDH Assay

An ALDEFLUORTM Kit (STEMCELL Technologies, Canada) was used for ALDH analysis in breast cancer cells following the manufacturer’s instructions. In brief, trypsinized single cells were suspended in the buffer containing the ALDEFLUOR substrate, incubated for 30 min at 37°C with or without the aldehyde dehydrogenase inhibitor DEAB, flowed by FACS analysis using a FACScan flow cytometer (BD Biosciences, United States). Data were analyzed with the FlowJo software.

### Mammosphere Formation Assay

Cancer stem cells were seeded into a 6-well ultra-low attachment plate (Corning, United States) with a density of 3,000 cells/well, and cultured in DMEM/F12 containing 1xB27 supplement (Invitrogen), 20 ng/mL of human epidermal growth factor (EGF; Sigma) and 20 ng/mL of human basic fibroblast growth factor (bFGF; R&D Systems) for 7–10 days. The mammospheres with diameter greater than 40 μm were counted under a microscope for quantitative analysis.

### Western Blot

The procedures for western blot analysis were the same as the procedure described by our previous publication (Xu et al., 2020). The primary antibodies (1:2,000) we used include: OCT4 (2750S, Cell Signaling Technology), NANOG (4903S, Cell Signaling Technology), KLF4 (ARG 55811, Arigo Inc.), SOX2 (3579, Cell Signaling Technology), h-TERT (sc-377511, Santa Cruz), DNMT1 (5032, Cell Signaling Technology), DNMT3A (20954-1-AP, ProteinTech), DNMT3B (57868, Cell Signaling Technology), α-Tubulin (ab7291, Abcam), and GAPDH (sc-47724, Santa Cruz). HRP-linked anti-rabbit IgG (7074S, Cell Signaling Technology), and HRP-linked anti-mouse IgG (7076S, Cell Signaling Technology) were used as secondary antibodies (1:5,000).

### TOP-FOP Reporter Assay

The TOP-LUC/FOP-LUC reporter structures were described previously (Wang et al., 2018). MCF-7 cells were seeded on 12-well plates at a density of 1 × 10^5^ cells/well. The next day, cells were co-transfected using lipofectamine 2000 (Invitrogen) with 0.5 μg of TOP or FOP reporter vector and 0.1 μg of pTK-RL plasmid. Twenty-four hours after transfection, the activities of *firefly* and *Renilla* luciferase were detected by AutoLumat using the Dual-Luciferase Reporter Assay kit (Promega, United States) following the manufacturer’s instructions.

### Gene-Based DNA Methylation Analysis

DNA was prepared from cells using a FastPure Cell/Tissue DNA Isolation Mini Kit (Vazyme, China). Conversion of methylated cytosine to uracil was mediated by bisulfite treatment using the DNA Bisulfite Conversion Kit (Tiangen, China). Methylation-specific PCR (MSP) primers were designed using Methyl Primer Express Software v1.0 (Epigentek Group Inc., Farmingdale, NY). PCR was performed using a T100^TM^ Thermal Cycler (Bio-Rad, United States).

### Genome-Based DNA Methylation Analysis

The MethylFlash^TM^ Global DNA Methylation (5 mC) ELISA Kit (EpiGenie, United States) was used to quantify the global DNA methylation status of cell samples. The fluorescence signals represents the methylation of DNA.

### *In vivo* Tumor Xenograft Model

Matrigel was mixed with 2 × 10^6^ MCF-7 cells and injected into the fat pat of the fourth mammary gland of a nude mouse (*n* = 16). The mice were separated randomly into two groups (*n* = 8 for each group), and tail vein-injected with magnetic nanoparticles carrying either anti-piR-823 mimics or anti-NC oligo (0.25 mg/kg body weight per dose every 2 days), respectively. The magnetic nanoparticles were prepared with the modified Zn_0.4_Fe_2.6_O_4_@SiO_2_ nanoparticles and the anti-piR-823 mimic oligo or anti-NC control oligo (2: 1 at room temperature). Immediately after each injection, a piece of magnet was placed near the fourth mammary gland for 1 h to enrich the nanoparticles into the tumor tissues. The volume of tumors was measured every 5 days until day 30 after cell transplantation when all the mice were sacrificed. Tumors were separated and subjected to further analysis.

### Statistical Analysis

Data are presented as mean ± SEM unless otherwise stated. Statistical significance was determined by a standard two-tailed Student’s *t*-test and one-way ANOVA followed by least-significant difference (LSD). *P* < 0.05 was considered statistically significant.

## Results

### Upregulation of piR-823 in Breast Cancer Cells and Cancer Stem Cells

**FIGURE 1:**
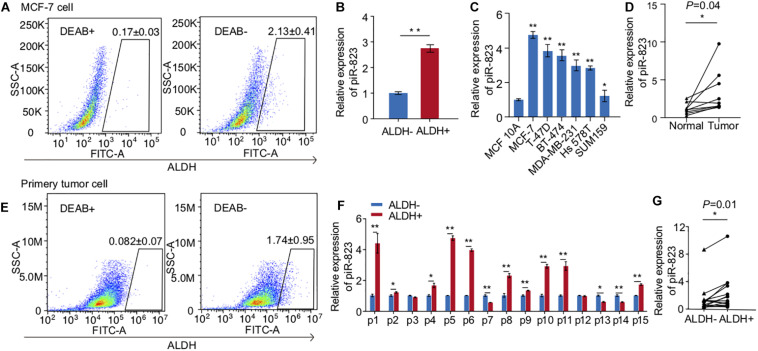
Upregulation of piRNA-823 in ALDH + breast cancer stem cells. **(A)** ALDH + CSC isolation from MCF-7 cells. **(B)** piR-823 showed upregulation in the ALDH + subpopulation of MCF-7 cells. **(C)** piR-823 expression analysis in different subtypes of breast cancer cell lines. **(D)** Upregulation of piR-823 in the tumor samples from luminal breast cancer patients, compared to the matched normal tissue from the same patient (*n* = 9). **(E)** ALDH + CSC isolation from breast cancer patients. **(F)** piR-823 expression analysis in ALDH + CSC from breast cancer patients (*n* = 15). **(G)** Statistical analysis of piR-823 expression levels in **(F)** Data are presented as mean ± SEM, *p < 0.05, **p < 0.01.

In view of the function of piRNAs in regulating germ cell stemness, DNA stability, and gene expression, we conducted analysis of piRNAs in regulating cancer cell stemness and gene expression. In order to address this question, we isolated ALDH + breast CSCs from MCF-7 human breast cancer cells (Figure 1A) for cancer-associated piRNA screening analysis, and identified that piR-823 was significantly upregulated in the ALDH + subpopulation of MCF-7 cells (Figure 1B). Additional analysis showed a higher level of piR-823 in the luminal subtype of breast cancer cell lines MCF-7, BT474, T-47D, and the basal-like subtype of breast cancer cell lines SUM159 and Hs578t, compared to the immortalized mammary epithelial line MCF-10A (Figure 1C). Nine pairs of clinical tissue samples (tumor and matched normal samples from the same patient) of luminal breast cancer were applied for piR-823 analysis, further indicating the upregulation of piR-823 in the tumors compared to normal tissues (Figure 1D). In addition, ALDH + breast CSCs isolated from primary tumor cells in breast cancer patients (Figure 1E) assessed for piR-823 analysis demonstrated the upregulation of piR-823 in CSCs from breast cancer patients (Figures 1F,G).

### piR-823 Promoted Cell Proliferation in MCF-7 and T-47D Lumianal Subtype of Breast Cancer Cells

In order to determine the biological function of piR-823 in breast cancer, targeted knockdown of piR-823 was conducted in MCF-7 cells (Figure 2A), resulting in decreased cell proliferation (Figure 2B) and colony formation (Figure 2C) *in vitro*. Similar results were obtained from another luminal subtype cell line T-47D (Supplementary Figures S1A,B). Consistent with this finding, piR-823 overexpression promoted cell proliferation in both MCF-7 (Figures 2D–F) and T-47D cells (Supplementary Figures S2A,B). These results suggest a proliferation-promoting role of piR-823 during tumorigenesis in the tested luminal subtype of breast cancer cells.

**FIGURE 2:**
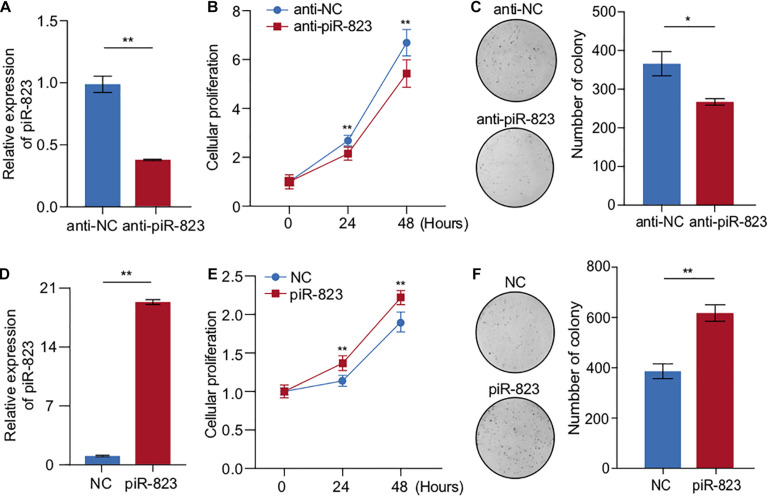
piR-823 promoted cell proliferation in MCF-7 and T-47D luminal subtype of breast cancer. **(A)** Validation of piR-823 knockdown in MCF-7 cells. **(B,C)** CCK8 assay **(B)** and colony formation assay **(C)** showing decreased cell proliferation by piR-823 knockdown in MCF-7 cells. **(D)** Validation of piR-823 overexpression in MCF-7 cells. **(E,F)** CCK8 assay **(E)** and colony formation assay **(F)** showing promoted cell proliferation in piR-823-overexpression in MCF-7 cells. Data are presented as mean ± SEM (*N* = 3), *p < 0.05, **p < 0.01.

### piR-823 Promoted Cell Stemness Acquisition in MCF-7 and T-47D Luminal Subtype of Breast Cancer Cells

**FIGURE 3:**
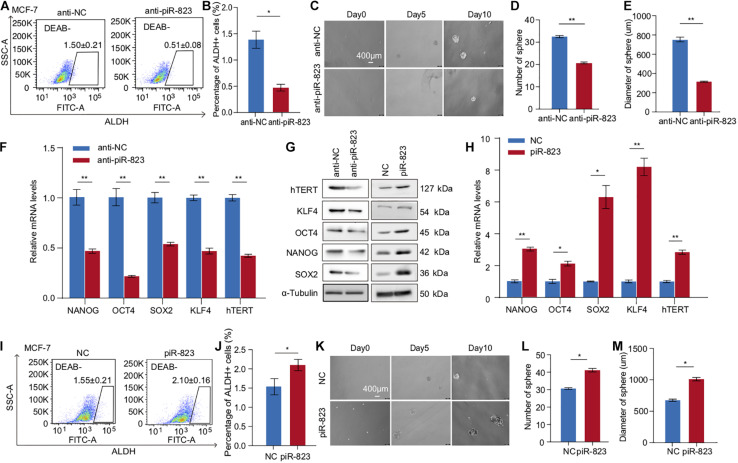
piR-823 promoted cancer cell stemness in MCF-7 and T-47D luminal subtype of breast cancer. **(A)** piR-823 knockdown in MCF-7 cells decreased ALDH + CSC subpopulation. **(B)** Quantitative analysis of **(A)**. **(C)** Mammosphere formation assays using MCF-7 cells with or without knockdown of piR-823. **(D,E)** Quantitative analysis of **(C)** showing decreased sphere number **(D)** and size **(E)** by piR-823 knockdown in MCF-7 cells. **(F–H)** Gene expression analyses showing positive regulation of stemness genes including OCT4, SOX2, KLF4, NANOG, and h-TERT at the mRNA **(F,H)** and protein **(G)** levels by piR-823. Knockdown or overexpression of piR-823 was applied in MCF-7 cells for the analyses. **(I)** piR-823 overexpression in MCF-7 cells promoted ALDH + CSC subpopulation. **(J)** Quantitative analysis of **(I)**. **(K)** Mammosphere formation assays using MCF-7 cells with or without overexpression of piR-823. **(L,M)** Quantitative analysis of **(K)** showing increased sphere number **(L)** and size **(M)** after piR-823 overexpression in MCF-7 cells. Data are presented as mean ± SEM (*N* = 3), *p < 0.05, **p < 0.01.

In order to determine the function of piR-823 in regulating CSCs in breast cancer, we analyzed the change in the ALDH + CSC subpopulation in MCF-7 cells after piR-823 knockdown (Figure 3A), indicating a decreased CSC proportion from ∼1.6 to 0.5% following knockdown of piR-823 (Figure 3B). Mammosphere assays demonstrated a significant decrease of both sphere number and sphere size after knockdown of piR-823 in MCF-7 (Figures 3C–E). In order to determine the mechanism governing the regulation of CSC by piR-823 in breast cancer cells, stem cell-regulating factors including OCT4, SOX2, KLF4, NANOG, and h-TERT were examined in MCF-7 cells after knockdown or overexpression of piR-823. The analyses demonstrated the induction of stemness gene mRNA expression and protein abundance by piR-823 (Figures 3F–H). Consistent with these findings, piR-823 overexpression in MCF-7 cells significantly promoted the percentage of the ALDH + CSC population (Figures 3I,J) and the capability to form mammospheres (Figures 3K–M).

In order to further validate the CSC-promoting function of piR-823, another luminal subtype of breast cancer cell line T-47D was assessed in breast cancer stemness assays including ALDH analysis and a mammosphere formation assay (Supplementary Figures S3A–E). The stemness genes OCT4, SOX2, KLF4, NANOG, and h-TERT were downregulated by anti-piR-823 and upregulated by piR-823 overexpression in T-47D cells (Supplementary Figures S3F,G). The results further showed that piR-823 participates in acquiring stem cell-like properties and/or maintaining CSC characteristics in the luminal subtype of breast cancer cells.

### piR-823 Promoted Mammary Gland Tumorigenesis *in vivo*

**FIGURE 4:**
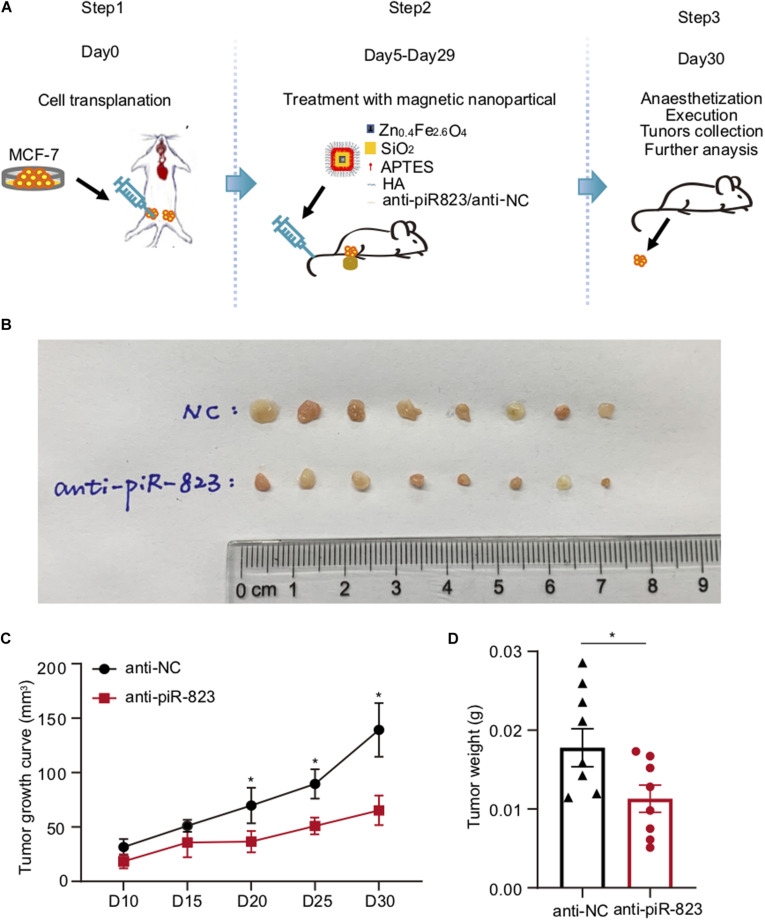
Tumor-targeted delivery of nanoparticles carrying anti-piR-823 suppressed breast tumor growth in a xenograft model. **(A)** Schematic representation of the procedure for the tail vein injection of Zn_0.4_Fe_2.6_O_4_@SiO_2_ magnetic nanoparticles carrying either anti-piR-823 or negative control anti-NC to the breast tumor-burdened mice transplanted with MCF-7 cells. **(B)** Tumor images isolated from the mice in **(A)**. **(C)** The tumor growth curves of the mice. **(D)** The weight of tumors in the mice. Data are presented as mean ± SEM (*N* = 8), *p < 0.05.

In order to determine the therapeutic potential of piR-823 in breast cancer treatment *in vivo*, a xenograft model was established using immunodeficient female nude mice by transplantation with the human breast cancer cell MCF-7, followed by a cancer cell-targeted delivery of magnetic HA-nanoparticles pre-loaded with anti-piR-823 oligonucleotides through tail vein injection (Figure 4A). On day 30 after cell transplantation, all the mice were sacrificed to obtain tumors (Figure 4B). The tumor growing curve showed a significant inhibition of tumor growth by anti-piR-823 treatment, compared to anti-NC control (Figure 4C), which was further supported by the weight of tumors in the two groups of mice (Figure 4D). All these data support the potential of piR-823 to serve as a therapeutic target to treat the luminal subtype of breast cancer.

### piR-823 Promoted DNA Methylation in MCF-7 Breast Cancer Cells

**FIGURE 5:**
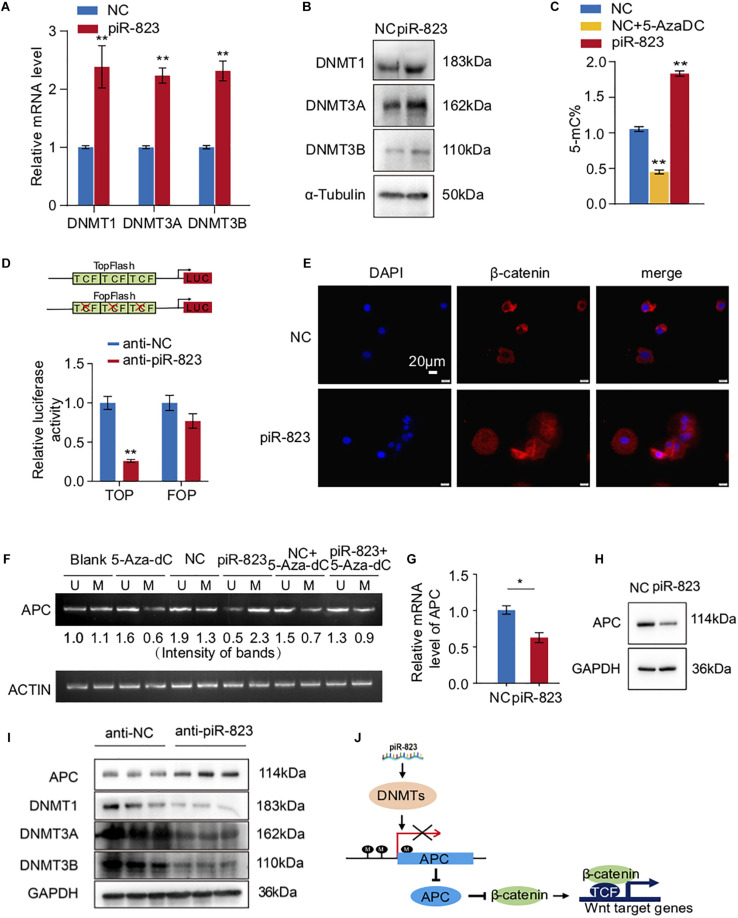
piR-823 activated Wnt signaling through regulating DNA methylation of APC promoter in MCF-7 breast cancer. **(A)** Upregulation of DNMT1, DNMT3A, and DNMT3B by piR-823 at the mRNA levels in MCF-7 cells. **(B)** Upregulation of DNMT1, DNMT3A, and DNMT3B by piR-823 at the protein levels in MCF-7 cells. **(C)** Increased global DNA methylation in MCF-7 cells by overexpression of piR-823. DNMT inhibitor 5-Aza-2’-deoxycytidine (5-AzaDC) was used as a positive control. **(D)** TOP/FOP assay demonstrated activated Wnt signaling by overexpression of piR-823. **(E)** Immunofluorescence staining assay indicating relocation of β-catenin from the cytoplasm to the nucleus in MCF-7 cells after overexpression of piR-823. **(F)** piR-823 promoted DNA methylation of gene APC promoter in MCF-7 cells. DNA methylation-specific primers were applied for PCR analysis to distinguish methylated and unmethylated APC. **(G,H)** Suppressed expression of APC at both mRNA **(G)** and protein **(H)** levels by piR-823. **(I)** Western blot analyses indicating the upregulation of APC and downregulation of DNMT 1, DNMT 3A, and DNMT 3B in the tumor samples of mice treated with anti-piR-823. **(J)** Schematic representation of the mechanism through which piR-823 promotes tumorigenesis in the luminal subtype of breast cancer by methylating the APC promoter and activating Wnt signaling, thereby regulating breast cancer stem cells. Data are presented as mean ± SEM (*N* = 3), *p < 0.05, **p < 0.01.

piRNAs have been reported to regulate gene expression via epigenetic mechanisms including regulating DNA methylation (Yan et al., 2015; Ai et al., 2019). We accordingly examined and detected the DNA methylation levels and DNA methyltransferase expression in the piR-823-overexpressing MCF-7 cells. As shown in Figures 5A,B, DNMT1, DNMT3A, and DNMT3B were upregulated after piR-823 overexpression in MCF-7 cells at both the mRNA and protein levels. We subsequently quantified the global DNA methylation status by specifically measuring the 5-methylcytosine (5-mC) levels in MCF-7 cells with or without piR-823 overexpression. The DNMT inhibitor 5-Aza-2′-deoxycytidine (5-AzaDC) was used to indicate the changes of DNA methylation level. As expected, upregulated 5-mC was associated with piR-823 overexpression in MCF-7 cells, which was attenuated by application of 5-AzaDC (Figure 5C).

### piR-823 Activated Wnt Signaling by Methylating the APC Promoter in MCF-7 Breast Cancer Cells

In view of the crucial function of Wnt signaling in regulating stem cell-like properties in cancer, we attempted to determine whether piR-823 had any effect on Wnt signaling. TOP/FOP flash luciferase reporter assays which serve as targets of Wnt signaling, demonstrated the decrease of Wnt activity by knockdown of piR-823 (Figure 5D), indicating a positive regulation of Wnt signaling by piR-823 in breast cancer cells. β-catenin, as the downstream key gene of Wnt signaling, was analyzed by immunofluorescence staining, indicating relocation from the cytoplasm to the nucleus in MCF-7 cells after overexpression of piR-823 (Figure 5E). Wnt signaling activity is frequently regulated by DNA hypermethylation in the promoter of Wnt/β-catenin pathway regulators (Tang et al., 2006; Samaei et al., 2014) including APC (adenomatous polyposis coli) which is under hypermethylation regulation at the CpG island region of its promoter during tumorigenesis (Esteller et al., 2000; Arnold et al., 2004). In order to determine whether it was APC hypermethylation that mediated piR-823-activated Wnt signaling, DNA methylation-specific primers were applied for PCR analysis to distinguish methylated and unmethylated APC at the DNA level. As shown in Figure 5F, hypermethylation of the APC promoter was induced by piR-823 overexpression in MCF-7 cells, which was diminished by application of the DNMT inhibitor 5-AzaDC. Associated with hypermethylation at the APC promoter region, piR-823 decreased APC expression in MCF-7 cells (Figures 5G,H). The regulation of DNMTs and APC by piR-823 were validated in the tumor samples from the xenograft model (Figure 5I).

## Discussion

Altered expression of piR-823 has been reported in various cancers including colorectal cancer (Yin et al., 2017), prostate cancer (Öner et al., 2016), and malignant breast cancer (Öner et al., 2016). Upregulation of piR-823 was induced after hormone treatment in prostate cancer cells and malignant breast cancer cells (Öner et al., 2016). In colorectal cancer, piR-823 was reported to promote cell proliferation and tumorigenesis by upregulating phosphorylation and transcriptional activity of HSF1 (Yin et al., 2017). In multiple myeloma, piR-823 promotes tumorigenesis by regulating *de novo* DNA methylation (Yan et al., 2015; Ai et al., 2019). However, piR-823 was demonstrated to suppress gastric carcinogenesis (Cheng et al., 2012). As such, piR-823 may display different expression patterns and different functional properties in different tumor types, playing a dual role in regulating tumorigenesis. Herein, we demonstrated the oncogenic function of piR-823 in association with the luminal subtype of breast cancer by regulating breast CSCs (Figure 5J).

There is only one prior publication studying piR-823 in breast cancer (Öner et al., 2016), in which external administration of estrogen increased piR–823 expression in MDA-MB–231 cells, while reducing piR–823 levels in MCF-7 cells. MDA-MB-231 cells are a basal-like subtype triple negative (ER, PR, and HER2 negative) breast cancer cell line whereas MCF-7 is a luminal subtype expressing both ERα and PR. These two cell lines have therefore different responses to hormone therapy. Although it is interesting that piR-823 responded to hormone treatment in breast cancer cells in a cancer subtype-specific manner, the biological significance and molecular mechanism of piR-823 function in breast cancer remain to be determined. In the current study, the upregulation of piR-823 in breast cancer was shown. The higher levels of piR-823 were determined not only in breast cancer cells, but also in the luminal subtype of breast cancer cells compared to basal-like subtype cells (Figure 1C), as well as in the luminal subtype of breast cancer patients (Figure 1D). In combination with the findings in the literature (Öner et al., 2016), we propose that high expression of piR-823 contributes to tumorigenesis in luminal breast cancer. Reduction of piR-823 may be considered as an indicator of patients’ therapeutic response to hormone treatment.

It has been demonstrated that CSCs are the main source of drug resistance, cancer recurrence, and metastasis. Development of novel therapeutic strategies targeting CSCs will shed light on the campaign to conquer cancer. Herein, we identified a small non-coding RNA piR-823 as a new regulator of CSCs in luminal breast cancer. Targeted knockdown of piR-823 significantly inhibited cancer cell proliferation and tumor growth *in vitro* and *in vivo*. Our finding not only demonstrates piR-823 as a novel target to treat the luminal subtype of breast cancer, but also provides help to understand the role that piR-823 may play when hormone therapy is applied to prevent and treat breast cancer.

## Data Availability Statement

The original contributions presented in the study are included in the article/Supplementary Material, further inquiries can be directed to the corresponding author/s.

## Ethics Statement

The studies involving human participants were reviewed and approved by the Institutional Review Board (IRB) of Tongji University Shanghai East Hospital. The patients/participants provided their written informed consent to participate in this study. The animal study was reviewed and approved by the Institutional Animal Care and Use Committee of the Tongji University School of Medicine.

## Author Contributions

ZY and CL designed the project and wrote the manuscript. XD, YL, YG, JL, ZL, WM, and PL performed all experiments. XD and QZ did data analysis. RP involved in the revision and language editing. All authors contributed to the article and approved the submitted version.

## Conflict of Interest

RP holds ownership interests in LightSeed, Inc. RP holds ownership interests (value unknown) for several patents and submitted patent applications. The remaining authors declare that the research was conducted in the absence of any commercial or financial relationships that could be construed as a potential conflict of interest.

## Supplementary Material

Supplementary Figure 1 Knockdown of piR-823 suppressed cell proliferation in T-47D breast cancer cells. **(A)** Validation of piR-823 knockdown in T-47D cells. **(B)** CCK8 assay showing decreased cell proliferation by piR-823 knockdown in T-47D cells. Data are presented as mean ± SEM (*N* = 3), ^∗∗^*p* < 0.01.

Supplementary Figure 2 Overexpression of piR-823 promoted cell proliferation in T-47D breast cancer cells. **(A)** Validation of piR-823 overexpression in T-47D cells. **(B,C)** CCK8 assay showing increased cell proliferation in piR-823-overexpressing T-47D cells. Data are presented as mean ± SEM (*N* = 3), ^∗^*p* < 0.05, ^∗∗^*p* < 0.01.

Supplementary Figure 3 piR-823 promoted cancer cell stemness in T-47D breast cancer cells. **(A)** piR-823 knockdown in T-47D cells decreased ALDH + CSC population. **(B)** Quantitative analysis of A. **(C)** Mammosphere formation assays using T-47D cells with or without knockdown of piR-823. **(D,E)** Quantitative analysis of C showing decreased sphere number **(D)** and size **(E)** by piR-823 knockdown in T-47D cells. **(F,G)** The stemness genes OCT4, SOX2, KLF4, NANOG, and h-TERT were downregulated by anti-piR-823 **(F)** and upregulated by piR-823 overexpression **(G)** in T-47D cells. Data are presented as mean ± SEM (*N* = 3), ^∗^*p* < 0.05, ^∗∗^*p* < 0.01.
