# Supplementary figures and images for: piRNA-823 Is Involved in Cancer Stem Cell Regulation Through Altering DNA Methylation in Association With Luminal Breast Cancer
Source: Front Cell Dev Biol. 2021 Mar 15;9:641052. doi: 10.3389/fcell.2021.641052 (PMC8005588; doi:10.3389/fcell.2021.641052)

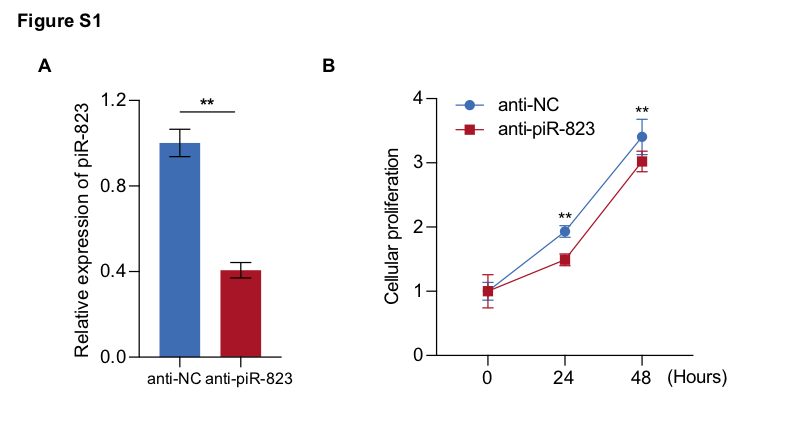

Supplement: Supplementary Figure 1 — Knockdown of piR-823 suppressed cell proliferation in T-47D breast cancer cells. (A) Validation of piR-823 knockdown in T-47D cells. (B) CCK8 assay showing decreased cell proliferation by piR-823 knockdown in T-47D cells. Data are presented as mean ± SEM (N = 3), ∗∗p < 0.01. [file Image_1.TIFF]

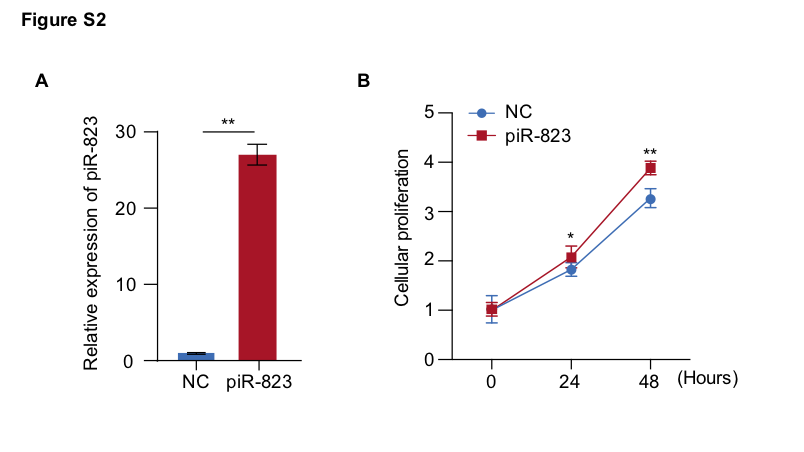

Supplement: Supplementary Figure 2 — Overexpression of piR-823 promoted cell proliferation in T-47D breast cancer cells. (A) Validation of piR-823 overexpression in T-47D cells. (B,C) CCK8 assay showing increased cell proliferation in piR-823-overexpressing T-47D cells. Data are presented as mean ± SEM (N = 3), ∗p < 0.05, ∗∗p < 0.01. [file Image_2.TIFF]

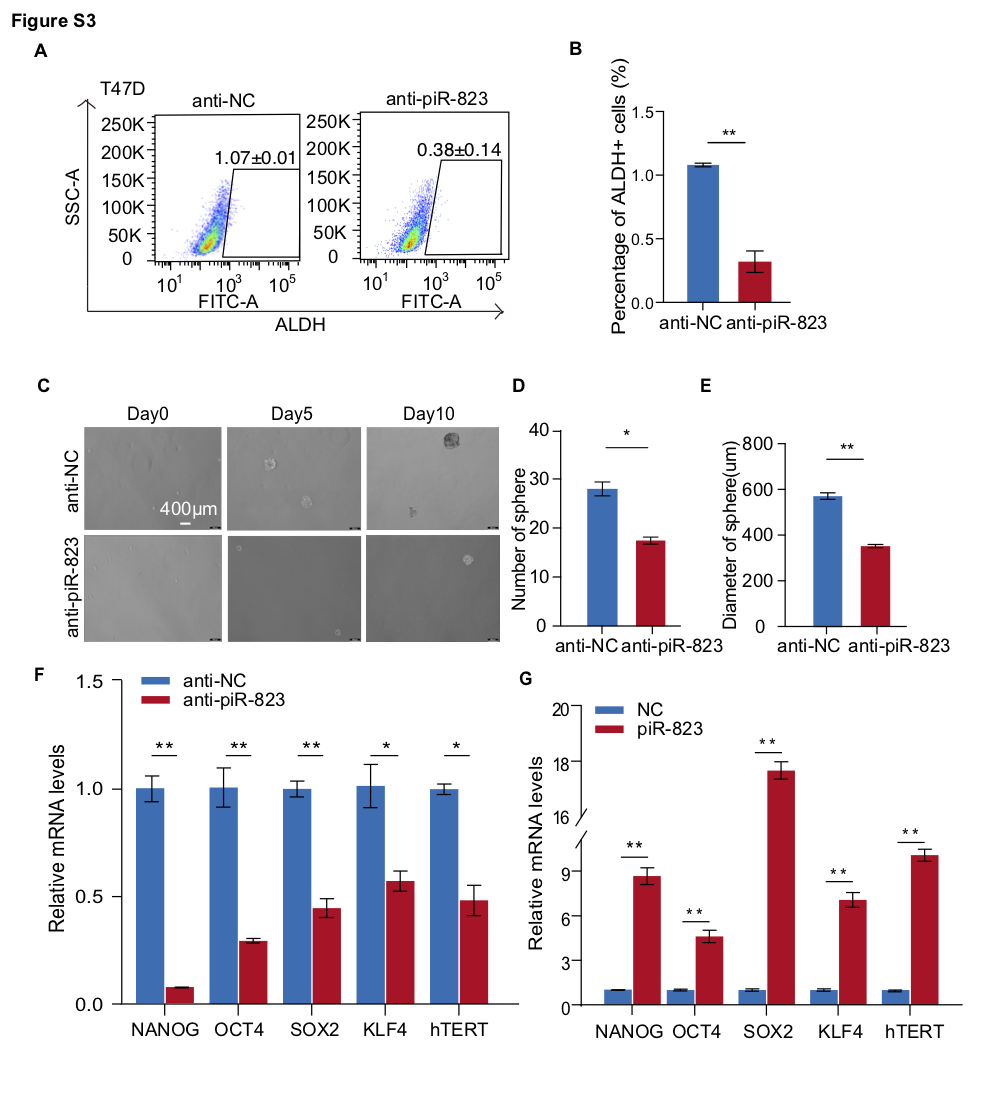

Supplement: Supplementary Figure 3 — piR-823 promoted cancer cell stemness in T-47D breast cancer cells. (A) piR-823 knockdown in T-47D cells decreased ALDH + CSC population. (B) Quantitative analysis of A. (C) Mammosphere formation assays using T-47D cells with or without knockdown of piR-823. (D,E) Quantitative analysis of C showing decreased sphere number (D) and size (E) by piR-823 knockdown in T-47D cells. (F,G) The stemness genes OCT4, SOX2, KLF4, NANOG, and h-TERT were downregulated by anti-piR-823 (F) and upregulated by piR-823 overexpression (G) in T-47D cells. Data are presented as mean ± SEM (N = 3), ∗p < 0.05, ∗∗p < 0.01. [file Image_3.TIFF]
